# Supplementary material for: Waste Polyurethane Foams as Biomass Carriers in the Treatment Process of Domestic Sewage with Increased Ammonium Nitrogen Content
Source: Materials (Basel). 2023 Jan 9;16(2):619. doi: 10.3390/ma16020619 (PMC9862140; doi:10.3390/ma16020619)
Supplement: Supplementary file 1 [file materials-16-00619-s001.zip › materials-2085013-supplementary.pdf]

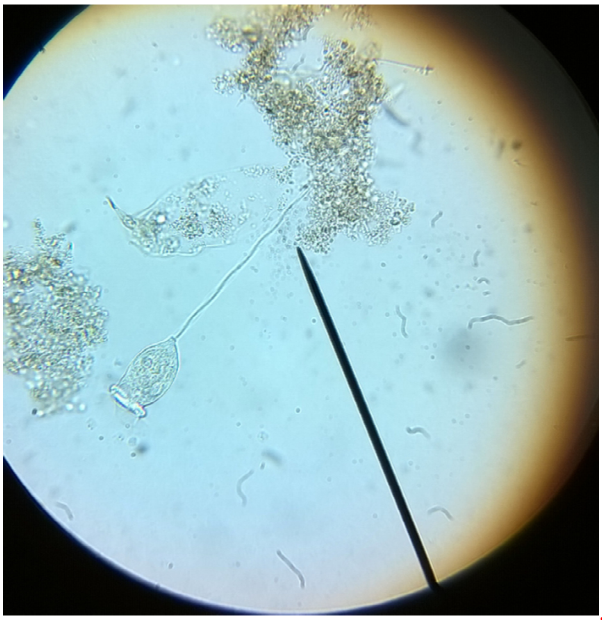

(**a**) Bluish-green foam, column A

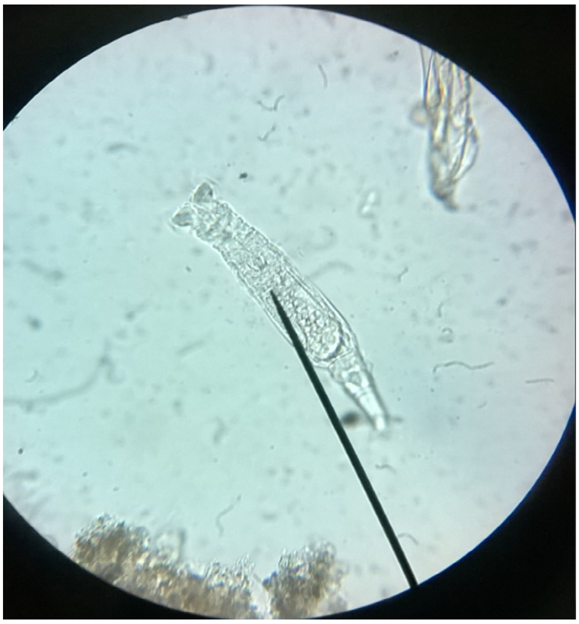

(**b**) Bluish-green foam, column B

Figure S1. Photo of (**a**) *Vorticella sp.*; (**b**) *Philodina sp*. (phot. E. Dacewicz).
